# Implementing the language comprehension test C-BiLLT: a qualitative description study using the COM-B model of behaviour change

**DOI:** 10.1186/s12913-022-08803-8

**Published:** 2022-11-28

**Authors:** J. N. Bootsma, M. Phoenix, J. J. M. Geytenbeek, K. Stadskleiv, J. W. Gorter, S. Fiske, B. J. Cunningham

**Affiliations:** 1 School of Rehabilitation Science, McMaster University, Hamilton, Canada; 2 CanChild Centre for Childhood Disability Research, McMaster University, Hamilton, Canada; 3 Amsterdam UMC Location VUmc, Amsterdam, Netherlands; 4 University of Oslo, Oslo, Norway; 5 Oslo University Hospital, Oslo, Norway; 6 Department of Pediatrics, McMaster University, Hamilton, Canada; 7 Pedagogical Psychological Services, Municipality of Oslo, Oslo, Norway; 8 School of Communication Sciences and Disorders, Western University, London, Canada

**Keywords:** Cerebral palsy, Communication, Assessment, Clinician behavior, COM-B model

## Abstract

**Background:**

It is challenging to reliably assess the language comprehension of children with severe motor and speech impairments using traditional assessment tools. The Computer Based instrument for Low motor Language Testing (C-BiLLT) aims to reduce barriers to evidence-based assessment for this population by allowing children to access the test using non-traditional methods such as eye gaze so they can independently respond to test items. The purpose of this study is to develop a contextualized understanding of the factors that influenced clinicians’ implementation of the C-BILLT in practice in the Netherlands and Norway.

**Materials and methods:**

A qualitative approach including semi-structured individual interviews with 15 clinicians (speech-language pathologists, neuropsychologists, and one teacher, counsellor, and vision specialist) was used. Data analysis was conducted in two rounds. First, a deductive approach including a codebook was used to code data within the COM-B components describing clinicians’ capability, opportunity, and motivation for behaviour change. Then, an abductive approach applying thematic analysis was used to identify meaningful patterns within the COM-B components.

**Results:**

Several meaningful barriers and facilitators were identified across the data. Clinicians used the C-BiLLT with two distinct groups of clients: (1) the population it was originally developed for, and (2) clients that could have also been assessed using a traditional language test. Clinicians working with the first group experienced more, and more complex barriers across all COM-B components, to successful C-BiLLT use than the latter.

**Conclusion:**

This study provides timely insights into the capability, opportunity, and motivation factors important for creating and sustaining assessment behaviour change in clinicians who used or attempted to use the C-BiLLT. Potential tailored intervention strategies aimed at improving implementation of novel assessment tools are discussed and may be helpful for others working to improve service delivery for children with complex needs.

## Background

Many children with cerebral palsy (CP) face barriers to communication [1–3]. Estimates vary, but it is thought that across all levels of motor functioning, 20–30% will have some difficulty expressing themselves verbally [4–6]. Of those with extensive motor impairments, it is estimated that 15–30% cannot use speech to communicate at all [4–6]. To alleviate the harmful impact of communication impairments on these children’s social, educational, and emotional well-being and development, Augmentative and Alternative Communication (AAC) interventions such as the introduction of graphic symbols, manual gestures, and speech generating devices are recommended [7–9].

Impairments in speech and expressive language production do not necessarily imply an impairment in comprehension [10], as a child’s understanding of spoken language can develop independently of their abilities to produce it [11]. This means children’s comprehension skills cannot be inferred based on their production skills. Each area of language must be independently assessed so AAC interventions can be developed and tailored to each child’s individual needs [4, 12, 13]. However, there are challenges associated with obtaining a reliable assessment of language comprehension in children with CP, because most tests require the participant to manipulate small objects, finger point, and/or speak [14]. As such, children with CP and limited motor or speech function are often excluded from standardized language assessments in both clinical practice and research studies [10, 15, 16], which may result in inequities in essential care, education, and opportunities to meaningfully participate. Instead of completing standardized testing for this population, clinicians may adapt components of standardized tests or estimate children’s language comprehension skills based on observation alone –unconventional methods that are unlikely to provide an accurate representation of children’s true skills.

The Computer-Based instrument for Low motor Language Testing (C-BiLLT) was developed to address the clinical and research gap regarding testing language comprehension in the CP population [17] (please see www.c-billt.com for more information). It facilitates an evidence-based (i.e., standardized, reliable and valid) assessment of spoken language comprehension for children who were previously often excluded from such testing. The test allows for a variety of response modes besides speaking, pointing, and object manipulation, and reliably assesses language comprehension in children with significant speech and motor impairments (for detailed descriptions of the psychometric properties of the C-BiLLT and its administration procedures, please see [12, 18, 19]). The C-BiLLT was first introduced in the Netherlands in 2014 and is included in the guidelines for the management of spastic CP [20]. In Europe to date, over 300 clinicians in the Netherlands, Norway, and Belgium have been trained to use the test. A recent study to assess implementation of the C-BiLLT in the European context surveyed clinicians working in various settings in the Netherlands and Norway. Clinicians rated the C-BiLLT highly on measures of acceptability, appropriateness, and feasibility, but they also identified several barriers associated with implementing it in practice [21].

The identification of barriers to implementation was not surprising as the uptake of new tools in practice is generally slow and use is low [22–25]. This is true despite knowledge that the use of evidence-based standardized assessment tools is considered integral to evidence-based, family-centred, and collaborative clinical decision making [26, 27]. Commonly-reported barriers to the use of new evidence-based assessment tools reported by clinicians include a lack of time, knowledge, and confidence in test selection and interpretation [28]; practical issues such as cost and ease-of-use; and a perceived lack of value in using tests [28, 29]. In addition to clinicians’ skills, perceptions, and beliefs, contextual factors such as work place setting and culture, managerial and organizational supports, and the wider health context can influence test adoption and use [30–32]. Barriers reported in the literature are similar to results reported in the aforementioned C-BiLLT implementation study, in which barriers were grouped into categories representing four factors: 1) inherent to the test, 2) related to the child, 3) related to the clinician, and 4) related to the environment [21]. While this initial implementation survey study was useful for identifying barriers and facilitators, a theoretically grounded approach is needed to fully understand clinicians’ behaviours. The removal of barriers alone may not be enough to predictably change clinician behaviour in the desired direction [33], presumably due to the many organizational and individual factors that interact to influence whether new tools are well implemented [33, 34].

The Capability, Opportunity and Motivation Behaviour Model (COM-B) [35] offers a framework for understanding behaviour in the context in which it occurs. The premise of the model is that for any behaviour to occur, there must be the capability to do it, the opportunity for it to occur, and a sufficiently strong motivation to carry it out. Capability, opportunity, and motivation are each divided into two types. As it relates to the C-BiLLT, psychological capability refers to a clinician’s knowledge, and cognitive, and interpersonal skills required for test administration. Physical capability refers to the skills needed to successfully operate the technology required to use the C-BiLLT. Opportunity can be physical, referring to the environmental opportunities (e.g., time, resources, physical barriers) that can facilitate or hinder use of the C-BiLLT, or social, referring to interpersonal influences, social cues and cultural norms associated with test use. Motivation covers the thought processes that direct behaviour. This can refer to clinicians’ reflective thought processes, plans and evaluations (e.g., clinicians planning to administer the C-BiLLT), or to automatic processes involving emotional reactions, desires, and impulses (e.g., clinicians feeling overwhelmed by something new). Components of the COM-B model are dynamic and interact over time, and a change in one component may lead to an increase or decrease in other components. For instance, increased motivation can lead people to do things that will increase their capability (e.g., taking a course) or opportunity (e.g., freeing up time). Similarly, increased opportunity or capability can increase motivation (e.g., you are more likely to do something if you know how and have the time to do it). Therefore, the COM-B model was used to understand the facilitators and barriers to tool use in the clinical context and what needs to shift or be modified to achieve desired behaviours [36]. This type of theoretical approach to implementation prevents an overreliance on educational approaches to overcome implementation barriers, which are known to have limited success [37, 38].

The European C-BiLLT implementation survey provided initial information about the complexities associated with implementing the C-BiLLT into practice [21]. The aim of this qualitative interview study was to better understand the factors that influenced clinicians’ implementation of the C-BILLT in practice by using the COM-B model. The new knowledge could be used as the foundation for further implementation efforts. The research question that guided the study was: How do capability, opportunity, and motivation influence clinicians’ use of the C-BiLLT?

## Methods

This study employed a qualitative descriptive design as presented by Sandelowski [39, 40]. In qualitative description, analysis remains close to the data when participants’ experiences are being interpreted so experiences can be comprehensively summarized using plain language.

Ethical approval for this study was obtained from the Office of Human Research Ethics at Western University. In the Netherlands, the study was classified as not subject to the Medical Research Involving Human Subjects Act (WMO), thus no additional ethical approval was required. In Norway, the Norwegian Centre for Research Data evaluated the ethical aspects of the study and gave permission for data collection (2020/# 967,236).

### Sampling and recruitment

The study was carried out in the Netherlands and Norway, the clinical contexts in which the C-BiLLT was introduced in 2014 and 2019 [12], respectively. Participants were recruited from the sample of respondents to our original survey [21]. Clinicians who participated in the survey study were recruited because they had previously met criteria for being trained to administer the C-BiLLT in Europe – this included primarily speech-language pathologists, but also psychologists and special education professionals [21]. After completing the original survey, clinicians were asked if they agreed to be contacted for a follow up interview, to which 33 clinicians agreed. As more clinicians agreed to follow up than were needed for the study, Dutch clinicians were purposefully sampled based on their experience administering the C-BiLLT (none, low 1–9 times, medium 10–20 times, high > 20 times). 14 Dutch clinicians were approached through email, with a maximum of two emails. Three Dutch clinicians did not respond, and two declined participation because of either a lack of time or a change of job (*n* = 9 were included). All Norwegian clinicians who volunteered to be interviewed (*n* = 6) were included. Because of the relatively small number of C-BiLLT trained clinicians in Norway, workplace details have been left out of the quotes for Norwegian participants to ensure their anonymity.

### Procedures and materials

Semi-structured interviews were conducted to understand clinicians’ experiences administering the C-BiLLT within their practice setting. The interview guide included introductory questions about clinicians’ professional background and current work setting, and four open-ended questions about the introduction, initial adoption, early implementation, and continued use of the C-BiLLT in their practice [41]. Interviews were conducted by two researchers who were also clinicians with experience using the C-BiLLT (JB and SF) in each participant’s preferred language (Dutch or Norwegian) and recorded using Zoom videoconferencing software to mitigate both geographic and pandemic-related challenges. No further contact was made with the participants after their interview. While it was necessary that interviewers have both clinical and C-BiLLT experience to ensure data were accurately captured and interpreted, it is possible that having clinical researchers as interviewers may have affected the collection and analysis. For example, participants may have answered more succinctly because they assumed a shared frame of reference between them and the interviewer.

### Data analysis

Data collection and analysis took place concurrently. Interview recordings were transcribed verbatim in either Dutch or Norwegian and then translated to English by team members who were fluent in both languages (JB, SF). Translation was required to ensure transcripts were in the same language for analysis, and to provide access to study data for all members of the international research team. Transcriptions and field notes on the interviews were imported into Dedoose [42]. After familiarization with the data by rereading transcripts and fieldnotes, analysis was conducted in two rounds. In the first round, a deductive approach to analysis was conducted [42] in which JB and SF first developed a codebook that included the six COM-B components and their definitions (i.e., psychological capability, physical capability, physical opportunity, social opportunity, reflective motivation, and automatic motivation). The codebook was piloted on the first seven interviews by JB and a trained graduate student research assistant. The codebook was subsequently updated with some clarifications about how the COM-B components manifested in relation to C-BiLLT use. For example, physical capability could manifest not only as the skill to connect multiple devices, but also as the agility, or lack thereof, to operate the C-BiLLT on the same device that was used by the participant. The remaining interview transcripts were coded according to the revised codebook. The extracted data in each COM-B component was identified as either a barrier or a facilitator to C-BiLLT use. In round two of data analysis, codes were grouped using an abductive approach [43] and analyzed using thematic analysis [44]. In an abductive approach, the researcher moves back and forth between data and theory, and makes comparisons and interpretations while searching for patterns. This allows the researcher to use pre-existing theories (e.g., the COM-B), while also remaining open and sensitive to the data and the possibility of new concepts, ideas and explanations [45]. Both the deductive and abductive approaches were important in the context of this study because the goal was to align results with components of the COM-B model (deductive), but also to remain open to clinicians’ experiences and perspectives within each component (abductive). Using a preexisting theory to support qualitative data analysis could lead to overlooking aspects of the data that do not fit the theory, but that may be meaningful to the research question [42]. This approach could also lead to overinterpretation of the data, if the data are forced onto the predefined theoretical concepts. These risks were mitigated (but cannot be fully excluded) by the second, abductive round of data analysis [42]. The research team most involved in data analysis (JB, MP, GH, KS, BJC) met monthly to discuss ongoing challenges. Visualizations of the codes and relationships between them were used to support discussions, and memos were used to record relevant discussions and coding notes. Throughout the analysis process, these frequent discussions helped to develop a nuanced and rich understanding of the data.

## Results

15 clinicians were interviewed between May and October 2021, and interviews lasted between 20 and 90 minutes. Characteristics of participants are described in Table 1.

**Table 1:** Characteristics of participating clinicians

	Netherlands (N = 9)	Norway (N = 6)
Sex		
Male	–	1
Female	9	5
Profession		
Speech-language pathologist	9	–
(Neuro-)Psychologist	–	3
Teacher^1^	–	1
Pedagogical psychological counsellor	–	1
Vision specialist	–	1
Practice setting		
School	–	2
Day care (children & youth) (neurodevelopmental disorders or acquired brain injury)	3	–
Early intervention setting for developmental language disorder	1	–
Congregate care facility	2	–
Pediatric rehabilitation	3	1
Adult rehabilitation	–	1
National competence center	–	2
Experience with the C-BiLLT		
None (0)	–	2
Low (1–9)	1	2
Medium (10–20)	3	2
High (> 20)	5	

Note C-BiLLT; Computer Based instrument for Low Motor Language Testing

^1^In Norway, special education teachers are certified to administer standardized tests under some circumstances

All 6 COM-B components were identified in the data and the identified barriers and facilitators within each COM-B component are listed in Table 2. A feature of the data was the difference in the type of clients that clinicians used the C-BiLLT with. There were clinicians who used the test with the population it was originally developed for (i.e., children with severe motor and speech impairments), but others reported using it with clients that could have also been assessed using a traditional language test but preferred the C-BiLLT’s touch screen. Identified differences between these two groups are discussed within the descriptions of the barriers and facilitators in each COM-B component as described below.

**Table 2:** Using the C-BiLLT in practice, barriers (−) and facilitators (+) experienced by all users, by clinicians using the C-BiLLT with the intended population, and by clinicians using the C-BiLLT in lieu of traditional language tests

	Capability		Opportunity		Motivation	
Factors Experienced by	Physical	Psychological	Physical	Social	Reflective	Automatic
All C-BiLLT users regardless of population or mode of assessment	+General assessment skills	+ Professional skill set (e.g., clinical reasoning, communication)	- Incompatibility of C-BiLLT software with organisation’s IT environment/policies	- Low status	+Evidence-based	**+**Agency
	- Insufficient practice				+ A smooth assessment	
Clinicians using C-BiLLT with intended population	- Lack of skills to accommodate for vision impairments during assessment	- Lack of knowledge re: vision impairments	- Equipment issues: lack of budget, lack of space, and/or lack of equipment		+ Makes it possible to “uncover what’s within”	**-** Technology related frustrations
	- Lack of skills to use eye tracking equipment	- Difficulty interpreting clients’ behaviour			+ Beliefs about positive consequences for clients	
Clinicians using C-BiLLT in lieu of traditional language tests			+ Easy to organize the assessment			

## Capability

Clinicians identified both facilitators and barriers associated with their capability to implement the C-BiLLT in practice.

Facilitators. All clinicians described themselves as having a strong professional skill set (psychological and physical capability), including clinical reasoning, general assessment, and communication skills to support their use of the C-BiLLT. One participant was the exception: she worked as a teacher and had a strong interest in AAC but no clinical background. She described how she felt “not completely confident” administering the test, and had consequently never administered it after participating in the training (NOR_06, C-BiLLT experience level: none).

Professional capabilities were described as sufficient for clinicians using the C-BiLLT with clients who had the manual abilities to participate in traditional language testing. In these cases, the C-BiLLT was often used with children who liked being able to only point or touch to give a response (versus manipulate small objects), and for those that struggled to await verbal instructions and/or to inhibit the urge to manipulate test materials. In these cases clinicians administered the test on an iPad or a touch screen laptop, so they did not need additional skills beyond the general technological skills needed to operate a computer or a tablet.

Barriers. Clinicians reported that the test became “technically more difficult” to perform if they had limited opportunities for practice (physical capability) (NOR_01, psychologist, C-BiLLT experience level: none). Reported reasons for lack of practice included an absence of eligible clients, a lack of necessary equipment, and measures associated with COVID-19 that restricted in-person assessments. In some cases, these barriers led to abandonment of the test.

Capability barriers were more complex for clinicians who administered the test with the population it was developed for (i.e., clients with severe motor and speech impairments). These clinicians used, or attempted to use several different access methods, which meant they needed skills in technology. Two main barriers were identified for clinicians using the C-BiLLT with its intended population: a) using the test with children who had visual impairments, and b) challenges associated with various technologies.

Clinicians reported a lack of sufficient knowledge about their clients’ visual impairments. For example, clinicians were aware of clients’ visual impairments, but were unsure about the nature of the visual impairments and whether or how they could impact C-BiLLT assessment results (psychological capability). Clinicians also reported being unsure how to accommodate for the visual impairments during testing (physical capability).

“Children with CP obviously have a very big risk or, a lot of CVI occurs. And so directing your gaze and being able to hold it for a while is sometimes very difficult. And processing the visual information … how does that work? And is that for your first item still the same as for item 12 when you get that far? Have you already deleted all the photos you’ve seen, or is it just piling up? I don’t know much about that.” (NLD_06, SLP working in pediatric rehabilitation service, C-BiLLT experience level: medium).

Visual impairment was also reported to cause uncertainty when interpreting a client’s behaviour during the assessment and thus made some clinicians doubt the validity of test results. It was hard for clinicians to judge “whether children gave the wrong answer because they didn’t see it or because they just really gave the wrong answer” (NLD_05, SLP working in a facility for adults with neurodevelopmental disorders or acquired brain injury, C-BiLLT experience level: medium).

Technology barriers experienced by this group of clinicians centred around eye-tracking equipment. They mentioned a lack of knowledge and skills needed to connect and operate eye tracking hardware and software: “I noticed that I had a lack of knowledge to, for example, make the mouse cursor disappear or make it invisible” (NLD_05).

## Opportunity

Opportunities afforded or denied by a clinician’s work environment (e.g., their organization, larger practice context, or colleagues) impacted the success of C-BiLLT implementation.

Facilitators. Clinicians that used the C-BiLLT with children who could have completed a traditional test, reported how easy it was to organize the assessment (physical opportunity). They liked not needing to coordinate the use of testing materials with colleagues, especially when the clinician could use their own tablet to administer the test. Administration via iPad or touch screen laptop eliminated the need for a sizeable assessment space that would be needed to spread out materials for traditional language tests. Clinicians described ease of administration using a tablet regardless of the testing environment. Finally, for these clinicians the C-BiLLT was freely accessible after completing the training course, because there was no requirement to purchase additional equipment or materials to administer the test (physical opportunity).

Barriers. A commonly reported physical opportunity barrier was incompatibility between the C-BiLLT requirements and clinicians’ organizational IT systems or policies. For instance, the C-BiLLT works best using Chrome or Firefox, but some clinicians’ work computers or tablets were only set up for Safari and they were unable to change this setting themselves.

A social opportunity barrier mentioned by many clinicians was the lack of other professionals’ awareness or familiarity with the C-BiLLT. This meant the C-BiLLT had ‘low status’ among other professionals (e.g., in education), which presented barriers to implementation in several ways. In the Netherlands, in order to access schools with specific supports for those with speech and language impairments, children needed a certain criterion score on a standardized test. Clinicians who wanted to use C-BiLLT test results to support a client’s admission reported the test was not (yet) approved by the COTAN Review System of Evaluating Test Quality (a Dutch agency that oversees the quality of diagnostic tests). In these cases, C-BiLLT test results were not considered valid for determining whether children met criteria for school admission: “they just won’t accept it by definition” (NLD_02, SLP working in a day care for children and youth with neurodevelopmental disorders or acquired brain injury, C-BiLLT experience level: high).

A second social opportunity barrier was encountered by some clinicians that worked in settings that had prescribed sets of tests to be administered for all clients that did not include the C-BILLT. In these contexts, clinicians wanting to administer the C-BiLLT were required to meet with the responsible care coordinator, which prevented some from using the test.

*Interviewer: What I’m still thinking about huh … Well, you say you’re pretty positive about the C-BiLLT […] what makes it that you usually still go for the Schlichting or for another test?*

*Yes, because that’s in that format here in healthcare programming. It [the C-BiLLT] is really something new.[…] So I am really limited by the organization then.” (NLD_07, SLP who recently switched from working in pediatric rehabilitation services to an early intervention day care for toddlers with suspected DLD, C-BiLLT experience level: high).*

Even in work settings where there were no restrictions regarding test use, clinicians reported the C-BiLLT may not be familiar to other professionals, which was sometimes given as a reason for choosing another test.

Clinicians who used or attempted to use a variety of access methods, experienced several physical opportunity barriers. Common barriers included a lack of money to purchase equipment and a lack of space in which to use the equipment. For example, some clinicians reported having to share space or equipment with colleagues, and some worked at multiple locations, which meant having to move equipment and identify multiple assessment spaces. Some clinicians reported having access to the equipment they needed, but that the equipment was sub-standard (e.g., an available but poorly functioning eye-tracker). Physical opportunity barriers were often reported to have a cascading effect. Consider the following illustrative interview excerpt in which a clinician described not having access to an eye tracking device or a separate monitor for the client due to lack of funding:

*Interviewer: Then you both work on the touch screen.*

*Yes. Then I make sure that I only put the mouse [away], so that the child really can’t reach that. And that’s how we, how we, how it usually goes.*

*Interviewer: Right. And how do you like that, or how, how is that going?*

*Well that works. Only if children are really more limited, then it is more difficult. Because then I just sit next to the child. And then I can actually not see eye gaze 100% beautifully. (NLD_06, SLP working in pediatric rehabilitation service, C-BiLLT experience level: medium).*

This excerpt illustrates how an initial opportunity barrier (lack of money), led to another opportunity barrier (lack of appropriate equipment), which in turn led to physical capability (lack of agility), and psychological capability (inability to reliably observe a client’s response) barriers. To address barriers associated with equipment, clinicians reported solutions they perceived as suboptimal. For example, one clinician partnered with a colleague for every assessment, so the client’s eye gaze could be observed from two viewpoints rather than purchasing eye gaze technology. Others had clients touch a non-touch computer screen while the clinician used the mouse to select the client’s response. Partner-assisted scanning was also mentioned by several clinicians as a work around to purchasing eye-gaze technology. Partner-assisted scanning is an assessment administration method where the clinician points to the different pictures on the screen in a systematic manner and asks the client to indicate when the clinician points to the correct response. This was not a preferred method, but one that clinicians reported resorting to at times.

## Motivation

Facilitators. None of the clinicians were obliged (e.g. by management) to adopt the C-BiLLT in their practice. Instead, they all chose to do so themselves and expressed independence and agency (automatic motivation) in choosing to use the C-BiLLT.

Clinicians expressed motivation to use the C-BiLLT because they viewed it as a scientifically sound test, and felt that it facilitated a smooth assessment process (reflective motivation). The test was viewed as facilitating assessment because a) there were not too many subtests or objects to keep track of, b) the scoring was automatic which allowed clinicians to pay more attention to the client, c) there were fewer distractions for clients relative to traditional language tests, and d) all clients were familiar with and excited by working on a tablet or a computer. Additionally, Norwegian clinicians described the C-BILLT as filling a practice gap and unmet need for a standardized language comprehension test with (recent) Norwegian language norms.

“The first time I used it on adults I was a bit skeptical and thought like […] Will it be too childish? But they did not react to that at all. So it worked!” (NOR_05, psychologist, C-BiLLT experience level: medium).

Clinicians who administered the test with clients who could have completed another traditional language assessment felt the C-BiLLT saved them time, hassle, and money, while providing an evidence-based assessment of their clients’ language comprehension skills.

Clinicians who used the C-BiLLT with clients who had severe motor and speech impairments appreciated that the test allowed them to “uncover what’s within” their clients with more certainty compared to clinical observation alone (reflective motivation).

“Yes, I think there are a lot of kids who are locked-in. And um, um I can say that so many times, but if you see it in black and white as a caretaker or as a manager or remedial educator or whoever, that’s just a piece of evidence, of hey, look, it just is. Period.” (NLD_02, SLP working in a day care for children and youth with neurodevelopmental disorders or acquired brain injury, C-BiLLT experience level: high).

Clinicians using the C-BiLLT with its intended population described the test as something that they had been waiting for, a way to address an urgent clinical need and “a big leap forward” (NOR_04, psychologist, C-BiLLT experience level: low). Clinicians also noted that the C-BiLLT made it possible to access their clients’ language comprehension, and they believed an accurate understanding of language abilities would have positive consequences for their clients. More specifically, clinicians reported that the results from assessment with the C-BiLLT led to feelings of happiness and doing right by the client.

“I had a client who was severely disabled, eh, and where in fact everything was always handed to her, over which she had little control. But what we found out through this assessment is that she can choose between two things. So that she can choose, for example, what she wants on her bread, or that she can choose like, I want to wear my red sweater or my green sweater today. Hey, those are small steps, but perhaps important for a client, for the feeling that they belong and that they can also make their own decisions. So, yes, we like that.” (NLD_04, SLP working in a day care for children and youth with neurodevelopmental disorders or acquired brain injury, C-BiLLT experience level: medium).

Barriers. Technical (physical opportunity or physical capability) barriers encountered by the group of clinicians using the C-BiLLT with its intended population prompted feelings of frustration, annoyance or uncertainty (automatic motivation), which could negatively impact their C-BiLLT use.

*Interviewer: Right, I’m just thinking about if you go to that room and the touch screen, or the pc eye, is not there or the touch screen is not working, how do you proceed?*

*Yeah then I’m really annoyed [laughs] < Interviewer: [laughs] I get that>. (NLD_08, SLP working in pediatric rehabilitation services, C-BiLLT experience level: high).*

## Discussion

This study aimed to develop a comprehensive understanding of the behaviour of clinicians who implemented or attempted to implement the C-BiLLT into their practice. The COM-B model was used as a theoretical framework, and 15 clinicians were interviewed about their experiences using the C-BiLLT in practice. The COM-B model consists of components that are dynamic and can interact over time. These interactions were identified in the sample of clinicians who participated in this study and could help to explain their reported use or non-use of the C-BiLLT.

Several meaningful barriers and facilitators were identified across the data. Clinicians appreciated the C-BiLLT for its scientific rigor and because it helped them to achieve a more streamlined assessment process compared to traditional tests. This finding was consistent with the high ratings of acceptability (e.g., ‘I welcome the C-BiLLT’, and ‘The C-BiLLT is appealing to me’) reported in the initial survey study [39]. These positive motivational factors are reasons for the clinician to want to use the C-BiLLT and are likely to support the uptake of the instrument [31, 35]. However, performing a certain behaviour not only depends on motivation, but also on whether or not one can do it (i.e., if one has the required capability and opportunity). The commonly reported barriers (i.e., insufficient practice, incompatibility between the C-BiLLT and organizational IT, and a low status among other professionals) likely impacted the can-do aspect of C-BiLLT use. In our sample, the influence of these three barriers ranged from an inconvenience that could be dealt with (e.g., making time to call the IT department for assistance to install another web browser) to a reason to choose another test (e.g., equipment issues).

The type of clients a clinician used or wanted to use the C-BiLLT with (i.e., with the intended population or with any client) was an important distinction in our data because these groups had different experiences implementing the C-BiLLT.

For the group of clinicians that used the C-BiLLT with clients whom they could have assessed with another test, the benefits of using the C-BiLLT were clear. Once trained, these clinicians did not need additional skills (capability) or equipment (opportunity) because they could administer the test on a tablet, laptop, or computer they already possessed. For this group, the C-BiLLT removed a number of practical inconveniences they experienced with other tests. This pragmatic motivation came on top of the generally shared motivational factors about the test, which may have created a positive feedback loop where the clinician was highly motivated to use the C-BiLLT (i.e., wants to use it) and also had the capability and the opportunity to do so (i.e., can use it). Use of the C-BiLLT with populations other than the ones it was originally developed for is supported by findings from the survey study and highlights clinicians’ preferences for easy-to-use assessment tools [46].

The implementation experience was quite different for the group of clinicians using the C-BiLLT with its intended population. This group wanted to use the range of access methods compatible with the C-BiLLT (e.g., computerized eye tracking, input switches) to accommodate for their clients’ needs. Clinicians in this group described a more ideological motivation for wanting to use the C-BiLLT: the assumption that the test would help them to reveal the language comprehension abilities of their clients, which would then have positive consequences in their clients’ everyday lives. At the same time, these clinicians faced more capability and opportunity barriers to successful test use. The fact that many kept trying to address barriers and use the test illustrates the strength of their motivation.

The potential benefits of being able to assess this group of children with an evidence-based assessment tool are great. In research, the C-BiLLT could allow for more representative and inclusive samples in studies of language and cognition in children with CP, thus expanding the knowledge base for this population 10. In practice, individual children, their families, educators and treatment teams could benefit from a trustworthy assessment as a starting point to initiate, plan, monitor, and evaluate communication interventions. Together with the initial survey study [21], the results presented here demonstrate that despite these important benefits, barriers to C-BiLLT use remain and must be addressed in order to secure access to evidence-based language assessment for children with neurodevelopmental differences. Implementation interventions and future research should address the barriers experienced by clinicians, particularly those using the C-BiLLT with its intended population.

The COM-B model is at the hub of the Behaviour change wheel [47], which is a framework that can be useful for supporting the design of implementation interventions. More specifically, a COM-B analysis of current behaviours can be used to identify intervention strategies that are likely facilitate implementation. This theoretical approach can also help avoid less effective implementation strategies such as passive education-based strategies [48–50], or strategies that focus solely on the individual clinician [51], or the innovation [46] without considering the clinical context.

Insufficient opportunities for practice delay and jeopardize the innovation process, regardless of the reason(s) or cause(s) for the lack of practice. For example, in one survey study 685 nurses reported on their experiences with recently introduced technologies, and the right time frame between the training and availability of technology in daily practice was identified as an important factor for success [46]. This barrier requires a multi-factorial solution to ensure clinicians can maintain their knowledge and skills. In the current study, some clinicians reported a lengthy delay between when they received their C-BiLLT training, and when they were able to start using the test. If the lag is caused by organizational barriers (e.g., not providing funding for the equipment soon enough or an inability to quickly resolve the incompatibility issue), clinicians should be supported to prepare for C-BiLLT use even before they participate in the training. In the Netherlands, this is currently done by providing information about the technology requirements to everyone who enrolls in the training course. If the lack of practice opportunities stems from a lack of eligible clients or unforeseen challenges such as COVID-19 restrictions to in-person care, there should be opportunities for trained clinicians to practice their skills in the absence of ‘real’ test use. Opportunities could be offered virtually to accommodate clinicians’ schedules and to circumvent potential restrictions on in-person gatherings, and may include training videos to support general skills for C-BiLLT administration, as well as videos to support specific technical skills such as how to set up access methods like eye tracking equipment. In Norway, the lack of practice barrier was recently addressed by refresher webinar (November 2021), that from now on will be offered annually.

Education would be a meaningful strategy to address the knowledge barriers regarding vision impairments and their potential impact on C-BiLLT assessment, a barrier that was reported in the survey study as well [21]. Clinicians could be given additional information during the training course, or information could be available in the manual, or on the instrument’s website. Prompts or cues could be added to the C-BiLLT’s materials to direct clinicians reasoning about vision impairments before and during the assessment process, and when interpreting the results. For instance, users could be prompted to use the novel Eye-pointing Classification Scale [52], that was developed to describe looking behaviours in relation to eye-pointing in children with CP affecting their whole body using a five-point scale. This may help clinicians to reach a more precise description of a client’s behaviours during the C-BiLLT testing and to interpret the C-BiLLT scores with more confidence.

To address the impact of the C-BiLLT’s low status among other professionals and fields of practice, different actions could be taken. One is to engage in knowledge mobilization efforts to raise awareness about the C-BiLLT and its strong psychometric properties. For example, publishing about the C-BiLLT outside of academic journals and in media could raise awareness for professionals who are not familiar with the tool (e.g., professionals working in healthcare fields outside rehabilitation). Another one is to include the C-BiLLT as a measure in research. For example, the C-BiLLT was recently used in the Netherlands in a longitudinal study of language comprehension in children with CP [53], which has increased the C-BiLLT’s profile among participants and clinicians. Additionally, forthcoming results can be used as evidence for the C-BiLLT meeting the COTAN requirements for test quality, which would permit use of C-BiLLT test scores to support children’s admission to special schools. This research may also increase awareness about the test in the scientific and clinical communities over time. A final potentially impactful strategy for improving the C-BiLLT’s profile is to direct implementation efforts towards families in addition to providers, as such ‘patient-mediated’ knowledge translation interventions show promise in other healthcare domains [54].

In an attempt to resolve technology related barriers including feelings of frustration, easily accessible practical support could be offered. This could be achieved by a frequently asked questions section on the website, or by providing easy ways to contact developers and experts who can offer practical support. Lastly, to support clinicians who need to advocate for C-BiLLT resources (e.g., budget, space, equipment), documents could be prepared and distributed at or before training sessions. For instance, factsheets or infographics about the C-BiLLT may help individual clinicians to communicate the message that their client population requires an accessible language assessment instrument. Some of these types of practical supports have already been implemented in the Netherlands, in response to the feedback training participants.

### Strengths and limitations

A key strength of this study is that it presents a contextualized understanding of clinicians’ behaviour in assessment practices, an area of the literature with limited evidence to date [23, 51]. Paired with the quantitative data from the initial survey study, results have been used to identify meaningful barriers and facilitators to assessment instrument uptake in the real-word context [33].

One limitation of this study is the risk of social desirability bias, where respondents felt like they had to report positive aspects because they knew that members of the C-BiLLT team were involved in this project. This type of bias may also have influenced who agreed to enroll in the study. Judging from the results, however, participants appeared to feel comfortable sharing a variety of barriers. Still, it cannot be ruled out that this type of bias may have influenced who agreed to enroll in the current study, and who did not.

Another possible limitation is the imbalance in participants’ professional backgrounds as professional background, experience, and work setting may have impact on how they use the C-BiLLT and how they experience the assessment process. All of the Dutch clinicians were SLPs, while none of the Norwegian clinicians were SLPs. This difference was also present in the survey study, where all but one Dutch respondents were SLPs, and only one Norwegian respondent was an SLP. The C-BiLLT training is open to professionals who are trained to administer evidence-based psychodiagnostic assessment tools, regardless of their profession, but it is possible that those who meet this criterion differ by country or region. Similarly, it is possible that the clinical contexts in which the C-BiLLT might be administered differ outside of what we have learned about what is done in Norway and the Netherlands. The C-BiLLT is currently undergoing validation in several other countries, but it is important for teams to consider specific contextual factors when designing implementation plans.

## Conclusion

This study provided timely insights into factors important for creating and sustaining assessment behaviour change in clinicians who used or attempted to use the C-BiLLT. The novel use of the COM-B model identified complex interactions between the individual clinicians and their contexts. Future research should capitalize on this knowledge when designing implementation interventions. Future research will also explore the validation of the C-BiLLT as an assessment tool for other populations, such as children with other developmental conditions (e.g., Rett syndrome, Angelman syndrome), or adults with intellectual disabilities. In practice, clinicians wanting to implement the C-BiLLT should consider the identified barriers facilitators and attempt to address or mitigate barriers prior to moving forward.

## Data Availability

The data that support the findings of this study are available upon request from the corresponding author (JB). The data are not publicly available due them containing information that could compromise research participant privacy.

## Abbreviations

CP: cerebral palsy
CVI: cerebral visual impairment
SLP: speech-language pathologist
AAC: augmentative and alternative communication
DLD: developmental language disorder
C-BiLLT: Computer-Based instrument for Low motor Language Testing
COM-B Model: capability, opportunity, motivation behaviour model
COTAN: Dutch Review System of Evaluating Test Quality
Schlichting: Here used as short for the ‘Schlichting test for language comprehension’, a popular evidence-based Dutch test to assess language comprehension in children aged 2–7 years old
Mytyl/tyltyl education: Dutch schools for children with a physical disability and for children with both a physical and intellectual disability, respectively.
